# Interferon-α suppresses invasion and enhances cisplatin-mediated apoptosis and autophagy in human osteosarcoma cells

**DOI:** 10.3892/ol.2013.1762

**Published:** 2013-12-16

**Authors:** JUN ZHAO, MINGLI WANG, ZENG LI, JINGXIAN CHEN, ZONGSHEN YIN, JUN CHANG, DONGMEI GAO, SHIPING WANG

**Affiliations:** 1 Department of Parasitology, Xiangya Medical School, Central South University, Changsha, Hunan 410078, P.R. China; 2 Department of Microbiology, Anhui Medical University, Hefei, Anhui 230032, P.R. China; 3 Department of Pathology and Cell Biology, Columbia University, New York, NY 10032, USA; 4 Department of Orthopedics, The First Affiliated Hospital of Anhui Medical University, Hefei, Anhui 230032, P.R. China; 5 Department of Clinical Laboratory, The Third Affiliated Hospital of Anhui Medical University, Hefei, Anhui 230032, P.R. China

**Keywords:** interferon-α, apoptosis, autophagy, osteosarcoma cell, cisplatin

## Abstract

Interferon (IFN)-α is generated in response to viral infections and is used clinically in the therapy of a variety of viral infections and cancers. The present study investigated whether IFN-α could inhibit the invasive ability of osteosarcoma cells using a Matrigel invasion assay. In addition, the osteosarcoma cells were treated with cisplatin and/or IFN-α. Apoptosis and autophagy were assessed using 3-(4,5-dimethylthiazol-2-yl)-2,5-diphenyltetrazolium bromide assay, Hoechst 33258 staining, flow cytometry assay, acridine orange staining, green fluorescent protein-LC3 dot assay and transmission electron microscopy. Further analysis revealed that the efficacy of cisplatin was enhanced by the addition of the cytokine, IFN-α. These results indicate that the combination therapy of chemotherapeutics and IFN-α is a new approach for osteosarcoma, which requires validation by experiments *in vivo.*

## Introduction

Interferon (IFN)-α is a member of the type I interferon family, which is active as an antiviral and immunomodulatory cytokine. Type I IFNs are able to modulate a variety of cellular responses, including cell growth and apoptosis. In humans, the production of IFN-α is most efficiently induced in numerous types of immune cells by viral infection (1,2). IFN-α has been used clinically in the therapy of certain malignancies and viral diseases. Although the antitumor mechanism of IFN-α is not entirely understood, cell cycle arrest and apoptosis have been shown to be involved. These effects are likely to be independent of each other and are partly dependent on the cells that are treated with IFN-α (3,4). In particular, IFN-α has been shown to induce autophagy (5).

## Materials and methods

### Cell culture

The human osteosarcoma MG-63 cell line was obtained from the American type culture collection (Manassas, VA, USA). The MG-63 cells were propagated in Dulbecco’s modified Eagle medium (Gibco, Carlsbad, CA, USA) supplemented with 10% fetal bovine serum (Gibco) and antibiotics in a humidified incubator containing 5% CO_2_ at 37°C. Human IFN-α (Sigma-Aldrich, St Louis, MO, USA) was diluted in serum-free medium.

### Matrigel invasion assay

The Matrigel invasion assay was performed to assess the effects of IFN-α on the invasive properties of the MG-63 cells. Transwell inserts (12-well, 12-mm with 12.0-μm pore size) from Corning Inc. (Corning, NY, USA) were coated with 200 μl Matrigel (final concentration, 1.0 mg/ml in ice-cold serum-free medium; BD Biosciences, San Jose, CA, USA) and allowed to dry at 37°C for 3–5 h. The cells were treated for 48 h with IFN-α at concentrations of 1×10^2^, 1×10^3^ or 1×10^4^ IU/ml. The control and treated cells were washed twice with serum-free medium and trypsinized. A 200-μl cell suspension (2×10^5^ cells) from each sample was added to each well in triplicate.

The filters were incubated for 48 h at 37°C in a humidified incubator containing 5% CO_2_, fixed and stained with 0.5% crystal violet in methanol. The non-migratory cells were removed with a cotton tip and the migratory cells were counted using a light microscope (Olympus, Tokyo, Japan) at ×400 magnification. Each experiment was run in duplicate. The results are expressed as the mean number of cells that were counted in each field, ± standard deviation (SD).

### Cell proliferation assay

For the assessment of the cell growth, the MG-63 cells (5,000 cells/well) were treated with various concentrations of IFN-α and cisplatin in 96-well plates. At 48 h post-treatment, the cell growth was evaluated using a 3-(4,5-dimethylthiazol-2-yl)-2,5-diphenyltetrazolium bromide (MTT) assay. MTT (Sigma-Aldrich) was added to the culture medium in each well at a concentration of 500 μg/ml. Following incubation for 4 h at 37°C, 100 μl dimethyl sulfoxide (DMSO) was added to each well and the 550-nm absorption was measured. Each experiment was reproduced in six wells and repeated at least three times.

### Hoechst 33258 staining

The MG-63 cells were treated with various concentrations of IFN-α and cisplatin in six-well plates. At 48 h post-treatment, the cells were washed in cold phosphate-buffered saline twice and fixed in 4% formaldehyde at 4°C for 10 min. Following this, the fixed cells were washed and labeled with 5 μg/ml Hoechst 33258 (Sigma-Aldrich), and maintained at room temperature in the dark for 10 min. The cells were then observed and imaged using a fluorescence microscope (Olympus) with excitation at 350 nm and emission at 460 nm. Apoptosis of the MG-63 cells was determined by the alteration of nuclear morphology and fluorescent density that was observed subsequent to staining the cells with Hoechst 33258.

### Annexin V-fluorescein isothiocyanate (FITC)/propidium iodide (PI) double labeling for flow cytometry (FCM)-assessed apoptosis

An Annexin V-FITC kit (BD Biosciences) was used to detect apoptosis. The cells were cultured with various concentrations of IFN-α and cisplatin for 48 h, harvested through trypsinization and washed twice. The cells were then reacted with FITC-conjugated Annexin V and PI for 15 min. The staining profiles were determined using FACScan (BD Biosciences, Franklin Lakes, NJ, USA) and Cell-Quest software (BD Biosciences). The early apoptotic cells (Annexin-FITC-positive and PI-negative) were located in the lower right quadrant. The late apoptotic or necrotic cells (Annexin-FITC-positive and PI-positive) were located in the upper right quadrant. The healthy cells (negative for the two probes) were located in the lower left quadrant. The results are expressed as the percentage of positively-stained cells among total cells. Each group was repeatedly measured six times and each sample included 1×10^4^ cells.

### Acridine orange staining for autophagy

Autophagy is characterized by the formation and promotion of acidic vesicular organelles. The MG-63 cells were treated with various concentrations of IFN-α and cisplatin in six-well plates. At 48 h post-treatment, the cells were incubated with 1 mg/ml acridine orange (Sigma-Aldrich) for 15 min. Images were obtained using a fluorescence microscope.

### Green fluorescent protein (GFP)-LC3 dot assay

The cells were transiently transfected with a GFP-LC3 (Origene Tech., Inc., Rockville, MD, USA) vector using Lipofectamine LTX and PLUS Reagents (Invitrogen Life Technologies, Carlsbad, CA, USA) according to the manufacturer’s instructions. After 24 h, the cells were exposed to IFN-α and/or cisplatin for 48 h as indicated and examined under a fluorescence microscope. The induction of autophagy was quantified by counting the percentage of cells in each group that contained LC3 aggregates.

### Transmission electron microscopy

The cells were fixed with 3% glutaraldehyde in a 0.1-M cacodylate buffer for 1 h. Following fixation, the samples were post-fixed in 1% OsO_4_ in the same buffer for 30 min. Ultra-thin sections were then observed under a transmission electron microscope (JEOL, Tokyo, Japan).

### Statistical analysis

The experimental data are expressed as the mean ± SD. The group means were compared using a t-test via the statistical software program, SPSS 13.0 (SPSS, Inc., Chicago, IL, USA). P<0.05 was considered to indicate a statistically significant difference.

## Results

### IFN-α markedly reduces tumor cell invasion in MG-63 cells

**Figure 1:**
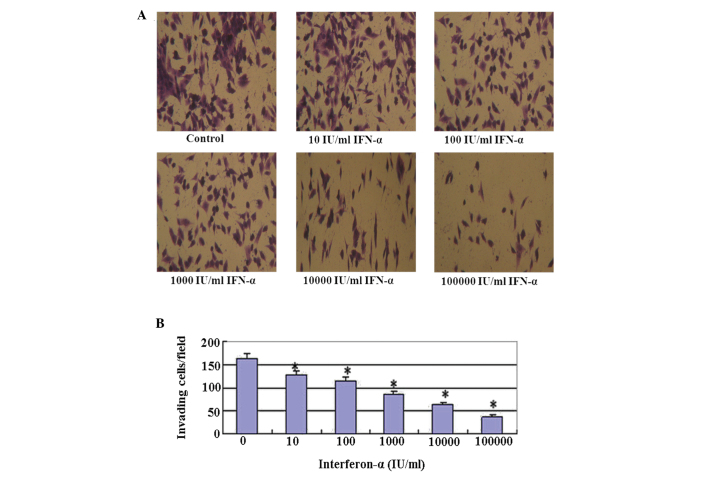
Cell invasion assay following treatment with IFN-α for 48 h. (A) Representative cell invasion assay for the MG-63 cells. The invasion assays were performed in 12-well Transwell inserts of polycarbonate filters with 12.0-μm pores that were coated with 200 μl 0.1% Matrigel. Following a 48-h incubation period, the membranes were collected and stained. A significant reduction in the number of invaded cells indicated a decrease in the invasive capacity (magnification, ×400). (B) Quantitative evaluation of the Matrigel invasion assay. The data are presented as the mean ± SD of 10 randomly selected microscopic fields from three independent wells (^*^P<0.01, compared with the control mean values). IFN, interferon.

Cell invasion was evaluated following the treatment (Fig. 1). Marked reductions in the invasive properties of the MG-63 cells were observed following the treatment with IFN-α alone through the Matrigel invasion assays. The staining of the invaded cells through the membrane demonstrated that the number of invasive cells was significantly reduced in the cells that were treated with IFN-α alone, compared with that of the untreated control cells.

### Anti-proliferative effects of IFN-α and/or cisplatin in MG-63 cells

**Figure 2:**
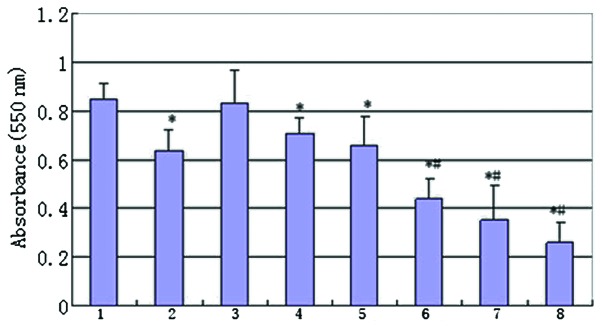
Antiproliferative effect of IFN-α and cisplatin. The MG-63 cells were treated with IFN-α and/or cisplatin. The cell viability was determined by an MTT assay. Group 1, control; 2, treated with 5 μg/ml cisplatin; 3, treated with 100 IU/ml IFN-α; 4, treated with 1,000 IU/ml IFN-α; 5, treated with 10,000 IU/ml IFN-α; 6, combination therapy of 100 IU/ml IFN-α and 5 μg/ml cisplatin; 7, combination therapy of 1,000 IU/ml IFN-α and 5 μg/ml cisplatin and 8, combination therapy of 10,000 IU/ml IFN-α and 5 μg/ml cisplatin. (^*^P<0.01, compared with the control mean values and ^#^P<0.01 compared with group 2 mean values). IFN, interferon; MTT, 3-(4,5-dimethylthiazol-2-yl)-2,5-diphenyltetrazolium bromide.

The effect of adding a low (100 IU/ml), middle (1,000 IU/ml) or high (10,000 IU/ml) dose of IFN-α on the antiproliferative activity of cisplatin was investigated. As shown in Fig. 2, cell proliferation was inhibited by exposing the cells to 100, 1,000 or 10,000 IU/ml IFN-α and/or 5 μg/ml cisplatin for 48 h. Cells treated with a combination of IFN-α and cisplatin exhibited more potent antiproliferative activity compared with those treated with cisplatin alone, in a dose-dependent manner.

### Effect of IFN-α and/or cisplatin on apoptosis in MG-63 cells

**Figure 3:**
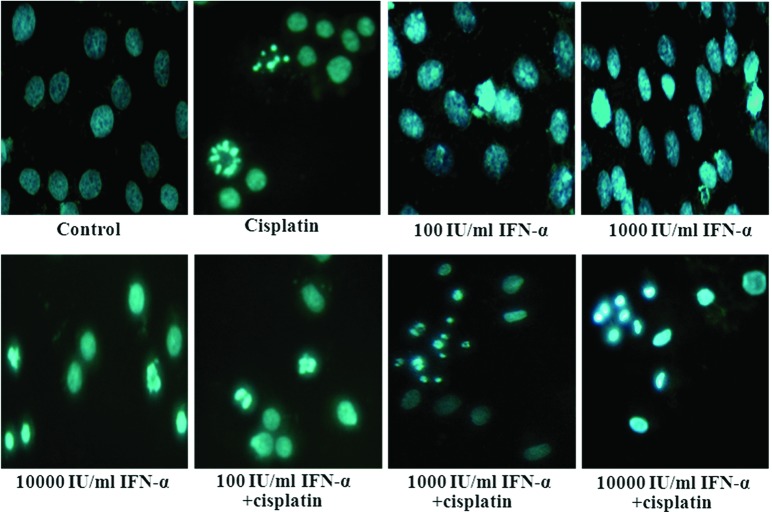
Hoechst 33258 staining to detect the of apoptosis of MG-63 cells induced by IFN-α and/or cisplatin. The MG-63 cells were incubated with various concentrations of IFN-α and/or cisplatin for 48 h prior to being stained with Hoechst 33258 (magnification, ×400). IFN, interferon.

**Figure 4:**
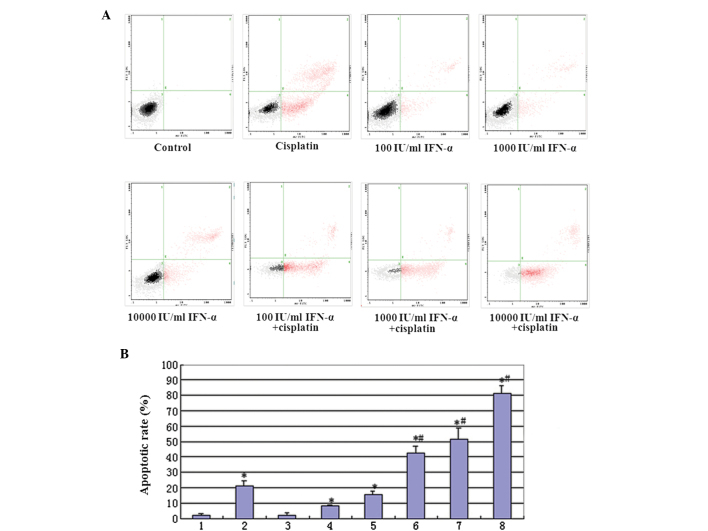
(A) Annexin V and PI staining for FACS detection of apoptosis of MG-63 cells induced by IFN-α and/or cisplatin. The MG-63 cells were incubated with various concentrations of IFN-α and/or cisplatin for 48 h prior to being stained with Annexin V and PI. (B) Quantitative evaluation of the FACS assay. Group 1, control; 2, treated with 5 μg/ml cisplatin; 3, treated with 100 IU/ml IFN-α; 4, treated with 1,000 IU/ml IFN-α; 5, treated with 10,000 IU/ml IFN-α; 6, combination therapy of 100 IU/ml IFN-α and 5 μg/ml cisplatin; 7, combination therapy of 1,000 IU/ml IFN-α and 5 μg/ml cisplatin and 8, combination therapy of 10,000 IU/ml IFN-α and 5 μg/ml cisplatin. (^*^P<0.01, compared with the control mean values and ^#^P<0.01, compared with treated with group 2 mean values). PI, propidium iodide; FACS, fluorescence-activated cell sorting; IFN, interferon.

The effect of adding a low (100 IU/ml), middle (1,000 IU/ml) or high (10,000 IU/ml) dose of IFN-α on the apoptotic action of cisplatin was investigated. The typical apoptotic morphological changes, including condensed chromatin and shrunken, crumpled and condensed nuclei, were observed in the MG-63 cells that were treated with IFN-α and cisplatin following staining with Hoechst 33258 (Fig. 3). A quantitative determination of MG-63 cell apoptosis that was induced by IFN-α and/or cisplatin was performed using Annexin V and PI staining. The cisplatin-mediated apoptosis effect, which was enhanced by IFN-α, was dose-dependent. The most evident apoptosis effect occurred following the addition of a high dose (10,000 IU/ml) of IFN-α and 5 μg/ml cisplatin (Fig. 4A). The quantitative results are shown in Fig. 4B.

### Effect of IFN-α and/or cisplatin on autophagy in MG-63 cells

**Figure 5:**
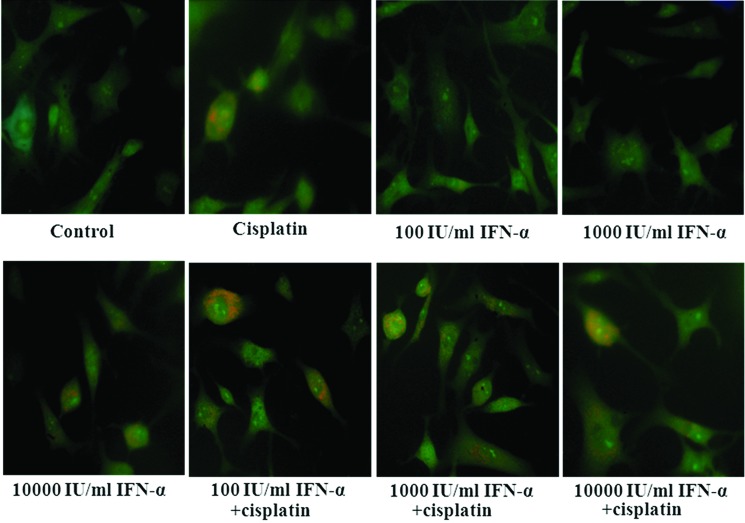
Acridine orange staining for autophagy. The cells were grown on coverslips, treated with IFN-α and/or cisplatin for 48 h and then stained with acridine orange. Autophagic vacuoles were observed and imaged using a fluorescence microscope (magnification, ×400). IFN, interferon.

**Figure 6:**
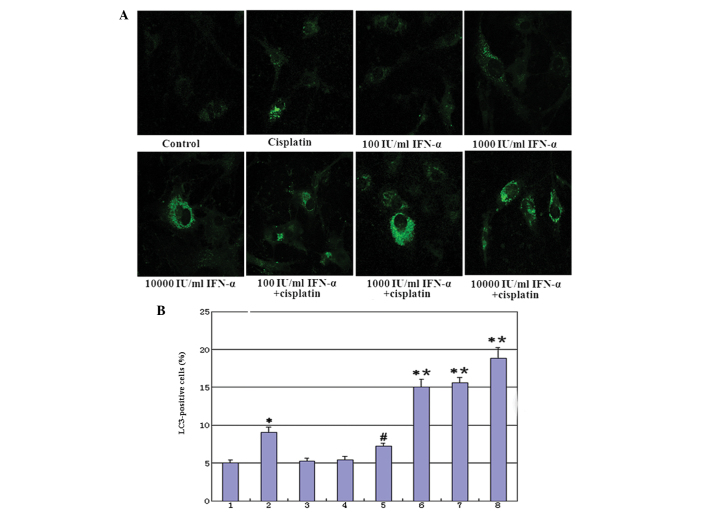
(A) IFN-α and/or cisplatin induced punctuated GFP-LC3 distribution in cells. At 24 h following the transient transfection of GFP-LC3, the cells were treated with IFN-α and/or cisplatin for 48 h and analyzed for fluorescence. The images were captured using a fluorescence microscope (magnification, ×400). (B) Quantitative assay: The induction of autophagy was quantified by counting the percentage of cells in each group that contained LC3 aggregates. Group 1, control; 2, treated with 5 μg/ml cisplatin; 3, treated with 100 IU/ml IFN-α; 4, treated with 1,000 IU/ml IFN-α; 5, treated with 10,000 IU/ml IFN-α; 6, combination therapy of 100 IU/ml IFN-α and 5 μg/ml cisplatin; 7, combination therapy of 1,000 IU/ml IFN-α and 5 μg/ml cisplatin and 8, combination therapy of 10,000 IU/ml IFN-α and 5 μg/ml cisplatin. (^*^P<0.01, compared with the control mean values; ^#^P<0.05, compared with the control mean values and ^*^P<0.01, compared with group 2 mean values). IFN, interferon; GFP, green fluorescence protein.

**Figure 7:**
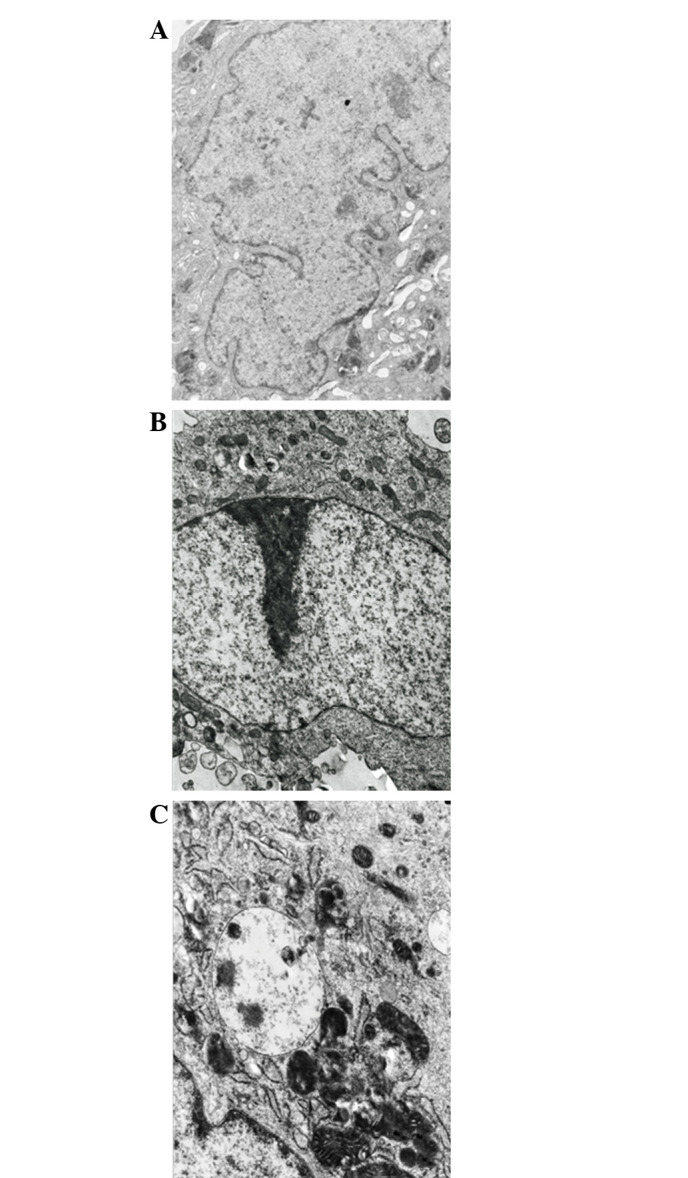
Transmission electron images of MG-63 cells that were treated with IFN-α and/or cisplatin. (A) Control group. (B) Combination therapy of 10,000 IU/ml IFN-α and 5 μg/ml cisplatin (group 8), chromatin margination and appearance of crescent-shaped chromatin. (C) Combination therapy of 10,000 IU/ml IFN-α and 5 μg/ml cisplatin (group 8). The arrow head indicates the single- and double-membrane vesicles containing intact and degraded cellular debris (magnification, ×6,000). IFN, interferon.

Following treatment with cisplatin or high-dose (10,000 IU/ml) IFN-α, the cells revealed an intracellular accumulation of acidic vesicular and autolysosomes, implying that cisplatin or a high-dose of IFN-α may induce autophagic responses. Co-treatment with IFN-α increased the cisplatin-induced acidic vesicular in the MG-63 cells. (Fig. 5). GFP-LC3 exhibited a diffuse pattern, which became punctuated when treated with cisplatin. Co-treatment with IFN-α increased the cisplatin-induced punctuated pattern in the MG-63 cells and the cisplatin-mediated autophagy effects were enhanced by IFN-α in a dose-dependent manner (Fig. 6A). The quantitative results are shown in Fig. 6B. Additionally, the co-treatment of IFN-α and cisplatin for 48 h developed autophagosome-like characteristics, including single- and double-membrane vacuoles containing intact and degraded cellular debris (Fig. 7). These data confirmed that the co-treatment with IFN-α increased cisplatin-induced autophagy in the MG-63 cells.

## Discussion

Osteosarcoma is the most common primary tumor of the bone and predominantly occurs in the second decade of life. The condition is the most frequently occurring pediatric non-hematological tumor of the bone and the fifth most prevalent malignancy of adolescence (6). Osteosarcoma is notable for locally aggressive behavior and early metastasis formation. High-dose cytotoxic chemotherapy and surgical resection have improved the prognosis of patients with osteosarcoma. The long-term survival for patients with localized (non-metastatic) disease is ~70%, but at the cost of considerable therapy-related morbidity (7,8). At present, ~20% of patients have metastases and almost all patients with recurrent osteosarcoma have metastatic disease. The cure rate for patients with metastatic or recurrent disease remains poor (9,10). Effective post-operative adjuvant therapies, therefore, are extremely significant for improving the overall survival of osteosarcoma patients.

IFN-α is a cytokine that belongs to type I IFNs and exerts multiple effects on cellular functions (1,11,12). The IFN-α family is composed of at least 13 functional IFN subtypes, which share the same receptor system and exert similar biological activities (13,14). In particular, 50 years of research on IFN-α have revealed that these cytokines exhibit a variety of biological effects, which differ from those that are present during viral replication, including antitumor activity. IFN-α is a widely expressed cytokine that is secreted as the first line of defense against several tumors (15). It has been used in >40 countries for the treatment of >14 types of cancer, including certain hematological malignancies (hairy cell leukemia, chronic myeloid leukemia and certain B- and T-cell lymphomas) and certain solid tumors, including melanoma, renal carcinoma and Kaposi’s sarcoma. However, despite numerous years of intense work in animal tumor models and considerable experience in the clinical use of IFN-α, the significance of the various mechanisms of action underlying the response in patients remains a matter of debate. It was previously considered that the direct inhibitory effects on tumor cell growth were the main mechanisms that were significant in the antitumor response in IFN-treated patients. However, it has been shown that IFN-α is able to directly inhibit the proliferation of normal and tumor cells *in vitro* and *in vivo,* and may exert other direct effects on tumor cells (16,17,18).

The present study on human osteosarcoma MG-63 cells demonstrated that IFN-α treatment suppressed human osteosarcoma cell invasion. The Matrigel invasion assays demonstrated marked reductions in the invasive properties of the MG-63 cells following treatment with IFN-α. The osteosarcoma cells were also treated with cisplatin and/or IFN-α. Apoptosis and autophagy were assessed using MTT assay, Hoechest 33258 staining, FCM assay, acridine orange staining, GFP-LC3 dotted assay and transmission electron microscopy. Further analysis demonstrated that the effects of cisplatin may be enhanced by combining the drug with IFN-α.

In conclusion, the present study has shown that IFN-α is able to suppress invasion and enhance cisplatin-mediated apoptosis and autophagy in human osteosarcoma MG-63 cells. The combination therapy of chemotherapeutics and IFN-α may be a novel approach for osteosarcoma, and further evidence is required by experiments *in vivo*.
