# [Expression of Concern] Interferon-α suppresses invasion and enhances cisplatin-mediated apoptosis and autophagy in human osteosarcoma cells

**DOI:** 10.3892/ol.2026.15885

**Published:** 2026-09-28

**Authors:** Jun Zhao, Mingli Wang, Zeng Li, Jingxian Chen, Zongshen Yin, Jun Chang, Dongmei Gao, Shiping Wang

Oncol Lett 7: 827–833, 2014; DOI: 10.3892/ol.2013.1762

Following the publication of this paper, it was drawn to the Editor's attention by a concerned reader that, regarding the cell invasion assay data shown in Fig. 1A on p. 828, the ‘100 IU/ml IFN-α’ and ‘1000 IU/ml IFN-α’ data panels were apparently matching, suggesting that this figure had been assembled incorrectly; moreover, the ‘Control’ and ‘10 IU/ml IFN-α’ data panels contained an overlapping section, suggesting that these data panels had been derived from the same original source.

The authors have been contacted by the Editorial Office to offer an explanation for these apparent anomalies in the presentation of the data in Fig. 1 of this paper, and we are awaiting their response. Owing to the fact that the Editorial Office has been made aware of potential issues surrounding the scientific integrity of this paper, we are issuing an Expression of Concern to notify readers of this potential problem while the Editorial Office continues to investigate this matter further.

